# An alternative posterior ascending pulmonary artery treatment in lobectomy with inflammatory lymph node infiltration

**DOI:** 10.1186/s13019-022-02033-z

**Published:** 2022-11-16

**Authors:** Yoshihito Iijima, Masahito Ishikawa, Shun Iwai, Aika Yamagata, Nozomu Motono, Sohsuke Yamada, Hidetaka Uramoto

**Affiliations:** 1 Department of Thoracic Surgery, Kanazawa Medical University, 1-1 Daigaku, Uchinada-Machi, Kahoku-Gun, Ishikawa 920-0293 Japan; 2 Department of Pathology and Laboratory Medicine, Kanazawa Medical University, Uchinada-machi, Ishikawa Japan

**Keywords:** Lung cancer, Inflammatory lymph nodes, En-masse lobectomy, Auto-stapler, Surgical resection

## Abstract

**Background:**

Lobectomy may be a challenging treatment option in lung cancer with inflammatory lymph node infiltration. Moreover, the en-masse lobectomy technique, which involves the simultaneous ligation or stapling of pulmonary vessels and bronchi at the hilar area, is controversial.

**Case presentation:**

We report the case of a 75-year-old woman who presented with lung cancer and lymph node infiltration from the posterior ascending pulmonary artery (A2) to the superior pulmonary artery (A6). A nodule was observed in her right upper lobe on chest computed tomography while treating her for a myocardial infarction 3 months prior; hence, a radical lobectomy was planned. Her main pulmonary artery could be constricted using surgical tape, but this was not possible in the peripheral pulmonary artery of the ascending A2 due to widespread lymph node infiltration. Intraoperative frozen sections confirmed the absence of metastases in the hilar lymph nodes. Pulmonary angioplasty was aborted because the cardiac function had not fully recovered from the previous procedure. The ascending A2 and upper lobe bronchus were collectively treated using an auto-stapler. Two months postoperatively, computed tomography showed no pulmonary artery aneurysm.

**Conclusions:**

This report highlights that the en-masse technique may be recommended as an alternative for A2 treatment during lobectomy in cases with inflammatory lymph node infiltration. Surgeons should consider switching to thoracotomy, in such cases, to avoid fatal intraoperative complications.

**Supplementary Information:**

The online version contains supplementary material available at 10.1186/s13019-022-02033-z.

## Background

Surgeons often encounter situations wherein total pneumonectomy must be avoided due to lung function or an underlying pre-existing condition. When inflammatory lymph nodes (LNs) infiltrate the pulmonary arteries (PAs) and veins, the risk of catastrophic bleeding increases, necessitating pulmonary arterioplasty or bronchoplasty. However, after considering the patient’s background characteristics, simultaneous ligation of the pulmonary arteries, pulmonary veins (PVs), and bronchi at the hilar region may be useful to reduce the surgical invasiveness. Essentially, in situations without metastasis to the hilar LNs, the management of the bronchi and pulmonary vasculature at the hilar region is central to a successful lobectomy. En-masse lobectomy, which is also called 'en-bloc' or 'tourniquet' lobectomy, has often been debated. It involves the simultaneous ligation or stapling of the pulmonary vessels and bronchi at the hilar region [1, 2].

## Case presentation

**Fig. 1:**
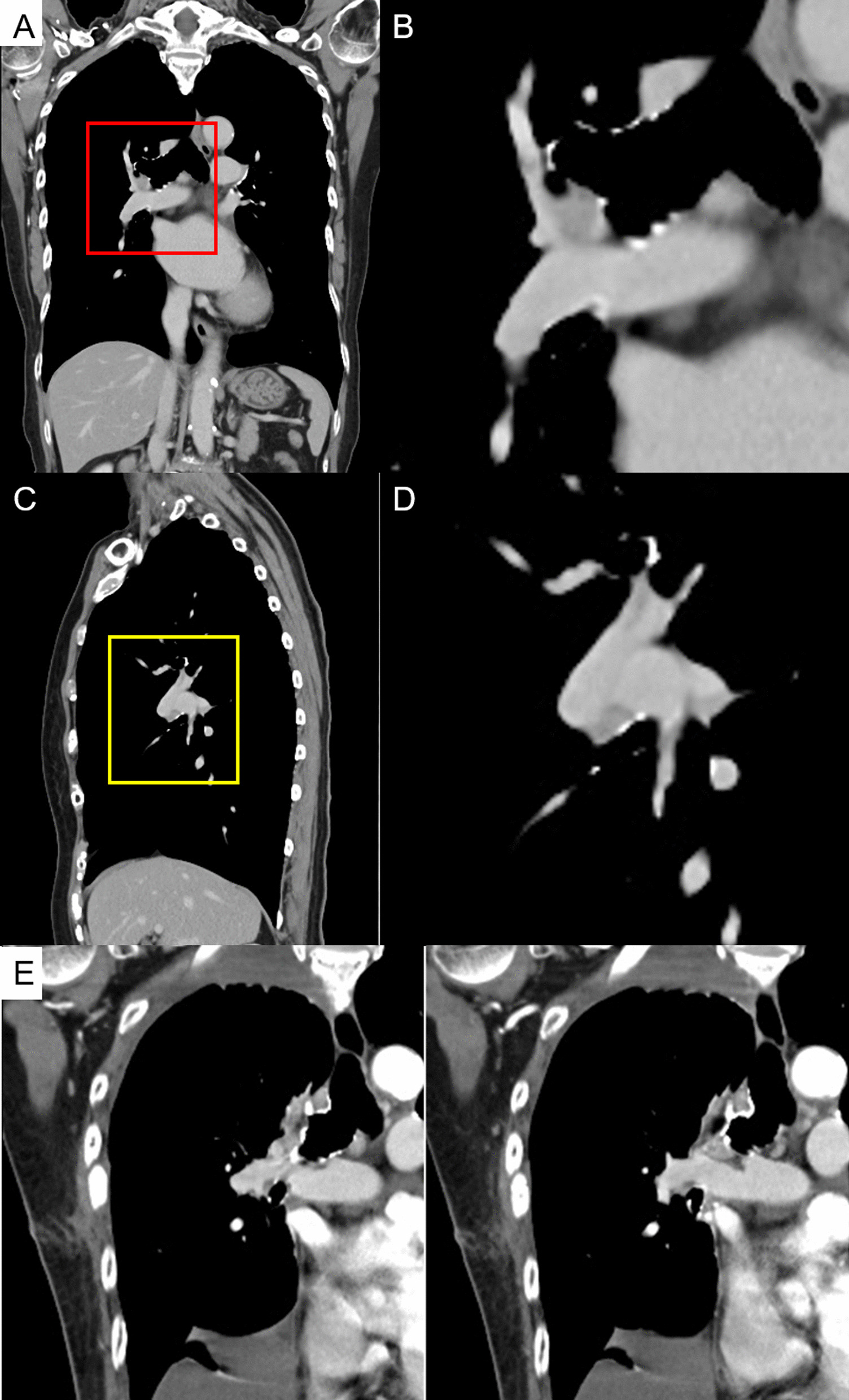
Chest contrast-enhanced computed tomography showing inflammatory lymph node infiltration of the hilar lymph node. **A**, **B** show the coronal views. **C**, **D** show the sagittal view. **B** is in the same range as the red square in (**A**). **D** is in the same range as the yellow square in (**C**). **E** Postoperative chest contrast-enhanced computed tomography showing no aneurysm in the A2 treated area

A 75-year-old woman experienced a myocardial infarction (MI) treated with percutaneous coronary angioplasty 3 months before her lung cancer surgery; therefore, dual antiplatelet therapy was started. Chest computed tomography (CT) performed while treating her for MI revealed a nodule in the upper lobe of the right lung. The interlobar LN (#11s) was surrounded by the posterior ascending PA (A2) and posterior bronchus (Fig. 1A, B) on one side and the superior PA (A6) and intermediate bronchial truncus on the other side (Fig. 1C, D), showing an unclear boundary with the PA. Therefore, inflammatory infiltration was suspected. Positron emission tomography–CT showed a maximum standardized uptake value of 2.20, indicating 2-deoxy-2-[^18^F]-fluorodeoxyglucose accumulation in the nodule of the right lung upper lobe without any significant accumulation in the hilar or mediastinal LNs. A right upper lobe lung cancer cT1bN0M0, stage IA2, was suspected. A preoperative evaluation of respiratory function was performed, which revealed no abnormalities. The stress electrocardiogram demonstrated a slight ST segment depression in the II, aVf, V5, and V6 leads. Echocardiography showed a normal left ventricular ejection fraction but abnormal wall motion in the septum, anterior wall, and apex region.

**Fig. 2:**
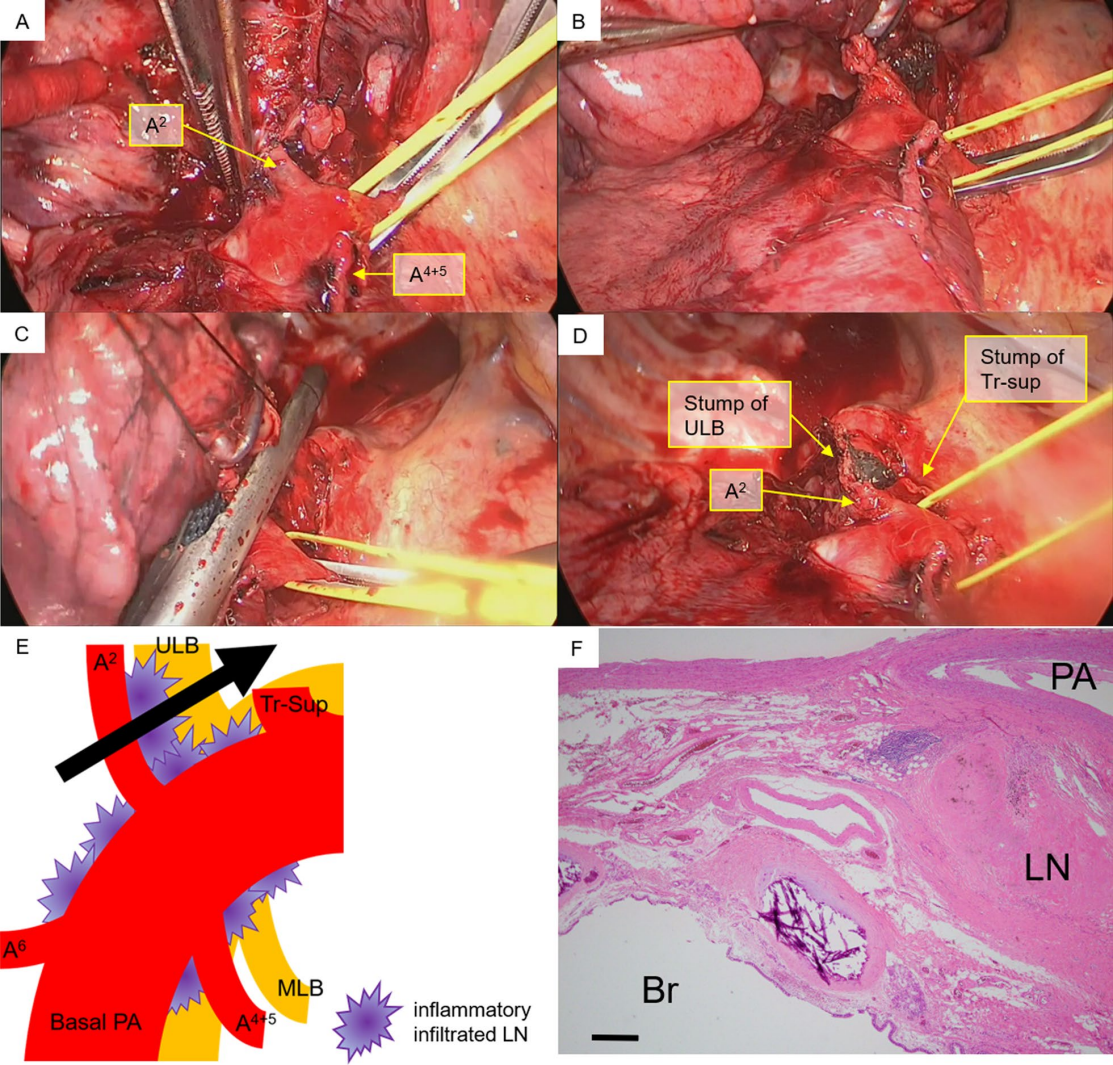
Intraoperative findings. **A**, **B** There was widespread inflammatory lymph node infiltration around the A2. **C**, **D** The A2 lesion coupled with the bronchus was treated using an auto-stapler (black; 4.2-mm cartridge). **E** The schema of treatment for A2 and the upper lobe bronchus. **F** No cancerous tissue was found at the bronchial stump, and a lymph node with silicotic nodules was found between the pulmonary artery and bronchus. Scale bar: 500 μm. LN, lymph node; ULB, upper lobe bronchus; Tr-sup, superior arterial trunk; MLB, middle lobe bronchus

The wedge resection for the nodule in the right upper lobe was performed by video-assisted thoracic surgery, and samples were submitted for intraoperative frozen section diagnosis. The patient was diagnosed with adenocarcinoma. Subsequently, a right upper lobectomy was performed. The upper lobe PV and superior arterial trunk were isolated using an auto-stapler. Moreover, there was widespread inflammatory LN infiltration around A2, which led us to suspect that detachment was impossible; thus, we converted to thoracotomy. After oblique fissure and minor fissure isolation, we attempted to detach the LNs around A2, but it seemed improbable; therefore, the main trunk of the PA was taped (Fig. 2A, B).

One of the hilar LNs (#10) was excised, and an intraoperative frozen section confirmed there was no LN metastasis. Blockade of blood circulation at the peripheral A2 was difficult due to inflammatory LN infiltration; therefore, pulmonary arterioplasty with PV clamping was considered. However, since this procedure would have been excessively invasive after MI, the A2 lesion coupled with the upper lobe bronchus (ULB) was secured using an auto-stapler (black; 4.2-mm cartridge) (Fig. 2C, D; see Additional file 1). The schema is shown in Fig. 2E. The bronchial stump (BS) was covered with free pericardial adipose tissue. The surgery required 183 min, and the estimated blood loss was 70 mL.

The postoperative course was uneventful; the chest tube was removed on the second postoperative day, and the patient was discharged on the ninth postoperative day. Histopathologically, the adenocarcinoma was diagnosed as pT1bN0M0, stage IA2. No cancerous tissue was found at the ULB stump, and an LN with silicotic nodules was found between the PA and bronchus (Fig. 2F). Chest contrast-enhanced CT performed 2 months postoperatively showed no aneurysm in the A2 area (Fig. 1E). Thereafter, the patient was maintained on regular follow-up through an outpatient clinic. No recurrence has been observed to date. Written informed consent was obtained from the patient for the publication of this report and its accompanying images.

## Discussion and conclusions

Preoperative evaluation of a patient to detect any inflammatory LN infiltration is central to preparing for pulmonary angioplasty and bronchoplasty. If any LN infiltration is preoperatively detected, converting to thoracotomy during thoracoscopic surgery is recommended to avoid catastrophic bleeding. Some studies have evaluated inflammatory LN infiltration using preoperative CT [3, 4]. Uramoto et al. [4] reported that preoperative contrast-enhanced CT of 5 mm slices could detect inflammatory LN infiltration based on the presence of an intervening adipose layer between the PA and LNs. In our case, an adipose layer was visible between the PA and LNs on the central side of A2, but it was unclear on the peripheral side. Moreover, no fat layer was found between the LNs surrounding A6, suggesting extensive inflammatory LN infiltration. Initially, A2 was considered treatable on the central side, and thoracoscopic lobectomy was attempted, but A2 could not be taped. Although the hilar region could be clamped for pulmonary angioplasty, taping the PA by retracting A2 was difficult because of the challenges in peeling around A6. We considered clamping the PVs of the lower and middle lobes, but 3 months after the treatment for MI, adverse effects on cardiac function were expected. A right upper en-masse lobectomy was performed after confirming the absence of LN metastasis by frozen section diagnosis of hilar LNs.

An en-masse lobectomy raises the following concerns [5]: intraoperative rupture of the PA stump, the risk of a bronchial pleural fistula (BPF) or bronchovascular fistula (BVF), and the curative value of the lung resection. Several technical details should be noted when en masse lobectomy is performed [6]: the surgeon must carefully dissect the target vessel and bronchus to ensure sufficient distance and space for placement of the auto-stapler, clamp the target at the thinnest part using the auto-stapler to ensure safe firing, use the thickest stapler cartridge, and shorten the stump and reinforce it to avoid postoperative bronchopleural fistula or bleeding. Additionally, surgeons are advised to switch to thoracotomy to avoid catastrophic intraoperative complications. Table 1 summarizes the cases of en-masse lobectomy for lung cancer reported in the literature. In all cases, the auto-stapler was used to process the lobe root structure. The cartridge used was green or black. When advanced auto-suturing devices are used, stapler failure rarely becomes an issue. Choosing the correct cartridge is essential. To the best of our knowledge, unlike a BPF, a BVF after en-masse lobectomy has not been reported [7]. Murakami et al. reported a microscopic examination of the stump with simultaneous bronchus and PA stapling 6 weeks after swine underwent lobectomy [8]. The stapled bronchial tissue remained as it was just after stapling, without degradation of the cartilage, smooth muscle, or epithelial layers. In contrast, the stapled PA tissue disappeared, resulting in the formation of a new vascular stump with recruitment of new intimal and adventitial layers and fibrotic tissue. Based in the results, they concluded that BVF will not develop after simultaneous bronchovascular stapling unless the anterior wall of the bronchus has fallen away due to major stump necrosis. Due to the different repair process, the BS was attached only by staples and covered with fibrotic tissue without histological fusion [8]. Therefore, more care was required to protect the BS. Herein, the BS was covered with free pericardial adipose tissue. Postoperative CT demonstrated the presence of intervening LNs between the PA stump and BS, while the risk of a BVF was considered low. Although no metastasis in the hilar LNs was confirmed, the possibility for insufficiency of LN dissection with the en-masse procedure remained. This patient required strict long-term follow-up.

**Table 1:** Summary of simultaneous stapling of the lobar root structure for lung cancer

Case	Age (years)	Sex	Histrogical type	Clinical staging	Surgical approach	Resected lobe	Treated vessels and bronchus	Procedure	Cartridge	Operation time (min)	Blood loss (g)	Pathological staging	References
1	69	M	ND	T1aN0M0, Stage I A	VATS	LLL	A6 + LLB	AS	Green	180	300	T1aN0M0, Stage I A	[1]
2	67	M	ND	T1aN0M0, Stage I A	VATS	RUL	A2 + ULB	AS + suture	Green	330	300	T1aN0M0, Stage I A	[1]
3	78	M	ND	T1aN0M0, Stage I A	VATS	LLL	A6 + LLB	AS	Green	210	Slight	T1aN0M0, Stage I A	[1]
4	77	M	ND	T1bN0M0, Stage I A	VATS	RML	A5 + V5 + MLB	AS	Green	150	Slight	T1aN0M0, Stage I B	[1]
5	66	F	Ad	ND	VATS	LUL	PVS + ULB	AS	Green	ND	ND	Stage I A	[4]
6	ND	ND	ND	ND	VATS	RLL	PVI + LLB	AS	Green	ND	ND	ND	[5]
7	75	F	Ad	T1bN0M0, Stage I A2	VATS → thoracotomy	RUL	A2 + ULB	AS	Black	183	70	T1bN0M0, Stage I A2	Our case

M, male; F, female; ND, not described; VATS, video-assisted thoracic surgery; LLL, left lower lobectomy; RUL, right upper lobectomy; RML, right middle lobectomy; LUL, left upper lobectomy; RLL, right lower lobectomy; LLB, lower lobe bronchus; ULB, upper lobe bronchus; MLB, middle lobe bronchus; AS, auto-stapler

In conclusion, the en-masse technique may be recommended as an alternative to A2 treatment in lobectomy with inflammatory LN infiltration. Surgeons should not consider switching to thoracotomy, in such cases, to avoid fatal intraoperative complications.

## Supplementary Information

**Additional file 1.** Intraoperative findings. There was widespread inflammatory lymph node infiltration around the A2, therefore, the main trunk of the PA was taped. The A2 lesion coupled with the upper lobe bronchus was secured using an auto-stapler (black; 4.2-mm cartridge).

## Data Availability

All data generated or analyzed during this study are included in this published article.

## Abbreviations

LN: Lymph node
PA: Pulmonary artery
PV: Pulmonary vein
MI: Myocardial infarction
CT: Computed tomography
A2: Posterior ascending pulmonary artery
A6: Superior pulmonary artery
ULB: Upper lobe bronchus
BS: Bronchial stump
BPF: Bronchial pleural fistula
BVF: Bronchovascular fistula

## References

[1] Kamiyoshihara M, Igai H, Ibe T, Ohtaki Y, Atsumi J, Nakazawa S (2013). Pulmonary lobar root clamping and stapling technique: return of the "en masse lobectomy". Gen Thorac Cardiovasc Surg.

[2] Lewis RJ, Caccavale RJ, Bocage JP, Widmann MD (1999). Video-assisted thoracic surgical non-rib spreading simultaneously stapled lobectomy: a more patient-friendly oncologic resection. Chest.

[3] Matsuura Y, Ichinose J, Nakao M, Okumura S, Mun M (2020). Prediction of and surgical strategy for adherent hilar lymph nodes in thoracoscopic surgery. Asian J Endosc Surg.

[4] Uramoto H, Nozu S, Nakajima Y, Kinoshita H (2015). Possibility of determining the degree of adhesion of the lymph node to the pulmonary artery preoperatively. J Cardiothorac Surg.

[5] Qiang G, Nakajima J (2015). Simultaneous stapling of pulmonary vein and bronchus in video-assisted thoracic surgery lobectomy. Ann Thorac Cardiovasc Surg.

[6] Liu C, Ma L, Pu Q, Liao H, Liu L (2016). How to deal with benign hilar or interlobar lymphadenopathy during video-assisted thoracoscopic surgery lobectomy-firing the bronchus and pulmonary artery together. J Vis Surg.

[7] Kamiyoshihara M, Ibe T, Kawatani N, Ohsawa F, Yoshikawa R (2016). Successful treatment of a bronchopleural fistula after en masse lobectomy. J Thorac Dis.

[8] Murakami J, Ueda K, Hayashi M, Sano F, Hamano K (2016). Simultaneous stapling of the lobar bronchus and pulmonary artery: is it actually dangerous?. Interact Cardiovasc Thorac Surg.

